# It Pays to Ask Twice: Alcohol Consumption Amongst Aboriginal and Torres Strait Islander Peoples Who Self‐Reported Not Drinking Alcohol

**DOI:** 10.1111/dar.70201

**Published:** 2026-07-16

**Authors:** Teagan Weatherall, James Conigrave, Scott Wilson, Jimmy Perry, Katherine M. Conigrave, Lynette Bullen, Taleah Reynolds, Dan Wilson, Monika Dzidowska, Martyn Symons, K. S. Kylie Lee

**Affiliations:** ^1^ Centre for Alcohol Policy Research La Trobe University Melbourne Australia; ^2^ Aboriginal Drug and Alcohol Council (SA) Adelaide Australia; ^3^ Faculty of Medicine and Health, Central Clinical School The University of Sydney Sydney Australia; ^4^ The Edith Collins Centre (Translational Research in Alcohol, Drugs and Toxicology), Sydney Local Health District Sydney Australia; ^5^ Drug Health Services, Royal Prince Alfred Hospital Sydney Australia; ^6^ Alice Springs Hospital, Northern Territory Health Alice Springs Australia; ^7^ Sydney Pharmacy School, Faculty of Medicine and Health The University of Sydney Sydney Australia; ^8^ National Drug Research Institute Curtin University Perth Australia; ^9^ Burnet Institute Melbourne Australia

**Keywords:** Aboriginal and Torres Strait Islander peoples, alcohol consumption, alcohol survey, Grog Survey App

## Abstract

**Introduction:**

It is important that Aboriginal and Torres Strait Islander peoples who experience worries from alcohol use are linked with support. Screening is important to help link people to support. However, individuals may not freely report consumption, especially if they perceive discrimination or drink less than others. Here we report on the use of a ‘check question’ asking about alcohol consumption on special occasions.

**Methods:**

We analysed data from an urban and remote community in South Australia, collected between July and October 2019. Participants who self‐reported not drinking alcohol were then asked, using a check question, if they had consumed *any* alcohol, including on special occasions. We explored the link between alcohol consumption and demographics using linear regression models. We used logistic regression to identify differences between individuals who did and did not initially self‐report alcohol consumption.

**Results:**

A total of 775 Aboriginal and Torres Strait Islander peoples participated. By using a check question, we identified 20 individuals who then self‐reported alcohol consumption in the previous 12‐months. They reported a median consumption of 5.4 standard drinks per occasion, had a lower AUDIT‐1 score and reported more consecutive days not drinking alcohol.

**Discussion and Conclusions:**

A check question in an alcohol survey tool was useful to identify individuals who do occasionally drink alcohol. Improving how screening tools ask about alcohol consumption is important to help people feel more comfortable and understand that the questions also apply to occasional drinking. This could help with collecting accurate data to inform prevention, treatment and policy efforts.

## Key Points

- Some Aboriginal and Torres Strait Islander peoples may self‐report not drinking alcohol if they only drink on special occasions.
- If an individual drinks only on special occasions, exceeding the recommended guidelines may still place them at risk of short‐term harm to themselves or others around them.
- Alcohol survey tools usually ask about frequency and intensity of usual or regular alcohol consumption and may not take into consideration drinking that occurs on special occasions.
- Using a ‘check question’ in an alcohol survey tool, such as on the Grog App, may better identify alcohol consumption patterns to inform healthcare, health promotion and policy needs.

## 1 Introduction

Aboriginal and Torres Strait Islander peoples have a long and valued connection to family, community, culture and Country [1, 2, 3, 4]. However, British colonisation caused the forced removal of Aboriginal and Torres Strait Islander peoples from their Country and the forced removal of children from families—the Stolen Generations. Aboriginal and Torres Strait Islander peoples have also endured systematic government efforts to remove their languages, social structures and culture [4, 5, 6, 7]. Alcohol use both stems from and compounds the effects of historical traumas that affect Aboriginal and Torres Strait Islander peoples [4, 8]. Alcohol is a major preventable cause of death, disease and broad socioeconomic problems, including for Aboriginal and Torres Strait Islander Australians [9]. Researchers, policymakers and communities need access to accurate screening data which can help identify and understand patterns of alcohol consumption and monitor the effects of efforts to reduce alcohol‐related worries.

The way that Aboriginal and Torres Strait Islander peoples approach alcohol surveys may be influenced by historical traumas, community or cultural norms, social comparisons, stigma or fear [10, 11]. For example, if people who drink alcohol (hereafter respectfully referred to as ‘drinkers’ to match our partner community's usage of the term and to improve accessibility) are asked if they are a ‘drinker’ it might remind them of stereotypes of, or compare themselves to, heavy drinking, instead of focusing on their own consumption [12, 13]. Questions about their drinking may then make them feel uncomfortable and less likely to report any occasional drinking. Additionally, existing survey tools such as Alcohol Use Disorders Identification Test‐Consumption (AUDIT‐C; a 3‐question alcohol screen) assume drinking regularity and might miss people who drink only a few times a year. Such factors should be accommodated for in survey design to enable accurate self‐reported drinking estimates amongst Aboriginal and Torres Strait Islander peoples, using culturally‐informed tools [14].

Some studies have asked about alcohol consumption in multiple ways, or cross‐checked answers via an Aboriginal worker. However, it has not been documented whether these efforts led to the reclassification of individuals to ensure accurate recording. In a 1992 study conducted in the remote Kimberley area in Western Australia by Hunter and colleagues, interviewers asked a series of questions to determine if participants drank ‘just around pay‐day’ or went for periods without drinking [15]. Similarly, in a 1991 study on drug use patterns by Fleming and colleagues in Northern Territory, an Aboriginal liaison worker cross‐checked some questions [16]. One solution is to use a ‘check question’, which asks individuals if they drink on special occasions. Occasions such as sporting or community events, or during ‘sorry business’ (periods of mourning after the death of a family or community member, or on the anniversary of a death).

While there are strong reasons to believe that check questions have utility in asking Aboriginal and Torres Strait Islander peoples if they drink alcohol, we are not aware of any published data on their effects. In this paper, we explore the utility of a ‘check question’ to identify alcohol consumption among Aboriginal and Torres Strait Islander peoples. In one urban and one remote South Australian community, we aimed to describe the proportion of self‐reported ‘non‐drinkers’ that did consume alcohol in the past 12 months, and the differences between individuals who self‐reported drinking alcohol compared with those who said that they consumed alcohol following the check question.

## 2 Methods

### 2.1 Ethical Approval

Ethical approval was obtained from the Aboriginal Health Council of South Australia (Reference: 04/15/621) and, as this was part of a larger study, from Metro South Health Queensland (Reference: HREC/16/QPAH/293).

### 2.2 Setting, Eligibility and Recruitment

The study was conducted in one urban and one remote community in South Australia. Participants were Aboriginal and/or Torres Strait Islander, aged 16 years or older and living in one of the two study sites. Data were collected between July and October 2019. In the urban community, 10 research assistants (seven Aboriginal and three non‐Indigenous; six men and four women) collected survey data. Participants were recruited in person through a range of community settings (e.g., public spaces like shopping centres, local events, primary care services, welfare, housing and related service providers, training, wellbeing and sporting programs). Quotas for age and gender were set ahead of time based on expected site demographics. Recruitment continued within each subgroup until the set quotas were filled, resulting in a sample with demographic characteristics closely matching those of the local population as reported in the most recent national census [17]. In the remote community, four research assistants (three Aboriginal and one non‐Indigenous; two men and two women) collected survey data. Due to the small number of eligible community members, we endeavoured to recruit all eligible participants. The project was promoted through local services and recruitment took place at planned events (e.g., barbeques at women's centre, council office, general stores) and ad hoc events in public spaces.

### 2.3 Data Collection

Data were collected as part of a 5‐year study that aimed to co‐create and validate a novel tool to help Aboriginal and Torres Strait Islander peoples describe any drinking (the ‘Grog Survey App’). The App collects self‐reported information on demographics (age, gender, income and employment status), alcohol consumption (modified Finnish method), money spent on alcohol, symptoms of alcohol dependence (ICD‐11), harms, access to treatment and user feedback. It has been shown to be an accurate and acceptable survey tool [18, 19, 20, 21]. Participants were asked about their last four drinking occasions because this method has been validated as a culturally appropriate way of capturing alcohol consumption amongst Aboriginal and Torres Strait Islander peoples [19, 20]. Participants also responded to a modified three‐item AUDIT‐C which used plain language and visualisations to improve accessibility [20]. The first item (‘AUDIT‐1’) was reworded to ‘Some people drink grog most days while others drink “once in a blue moon.” How often do you drink any grog at all?’. AUDIT‐2 visualised response categories in quantities of participants' most commonly consumed alcoholic beverage. AUDIT‐3 asked about the frequency that participants consumed more than 4 standard drinks to match national guidelines [22], with a visual depiction of 5 standard drinks. Participants were given headphones, the app read out questions in either English or Pitjantjatjara (an Aboriginal language commonly spoken in South Australia, Western Australia and the Northern Territory), in male or female audio. Participants entered in their answers independently, however research assistants were on‐hand to answer questions and help as needed.

### 2.4 ‘Check Question’ Used in the Grog App

**FIGURE 1:**
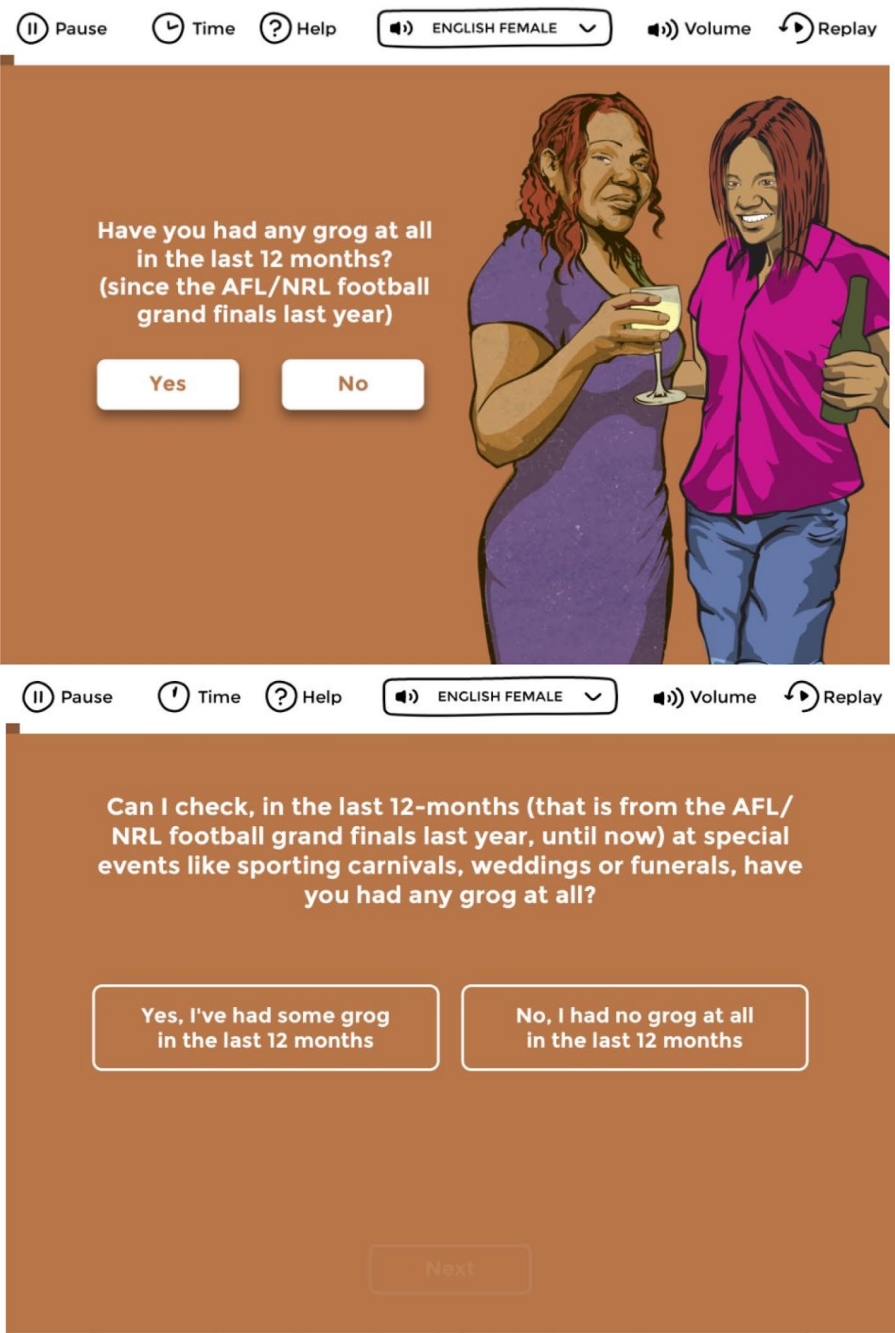
Screengrabs from the Grog App showing how people are asked about their alcohol consumption.

All study participants were asked: ‘Some people drink grog most days, while others drink “once in a blue moon.” How often have you had any grog in the last 12 months?’. Response options were: Never; Once in a blue moon (less than once a month); Sometimes (2–4 times a month); 2–3 times per week; Most days or every day. All participants who selected the response option ‘never’, indicating they did not drink in the last 12 months were then asked a ‘check question’. They were asked: ‘Can I check, in the last 12‐months, at special events like sporting carnivals, weddings or funerals, have you had any grog at all?’. Response options were: ‘Yes, I've had some grog in the last 12 months’ or ‘No, I had no grog at all in the last 12 months.’ (Figure 1). These responses led to unique survey pathways for individuals who responded with either ‘yes’ or ‘no’, respectively. For individuals who self‐reported drinking alcohol, consumption was then assessed using a modified ‘Finnish’ method [23] that asked in detail about the last two drinking occasions and the date of an additional two drinking occasions, in the last 12 months [20]. Note, the App calculates which reference point to use as a memory prompt (for ‘in the last 12‐months’ e.g., Easter holidays, New Years or football grand finals) and dynamically inserts these into the survey question.

### 2.5 Analysis

Data cleaning and analysis were performed in R (version 4.4.1) [24]. Urban and remote samples were combined. Age was recoded into four categories (Table 1). Highest level of education attainment was recoded into six categories (Table 1). Participants were classified into three risk categories based on their drinking behaviour: (i) low‐risk; (ii) risky but not likely dependent; and (iii) risky and likely dependent [21]. Participants who initially self‐reported as ‘non‐drinkers’ but later reported consumption via the special occasions check question were coded as ‘reclassified drinkers’.

**TABLE 1:** Demographics of reclassified drinkers and self‐reported current drinkers.

Variable	Reclassified drinkers, *n* = 20 (3.4%)	Self‐reported current drinkers, *n* = 577 (96.4%)
Remoteness		
Remote	1 (5.0)	42 (7.3)
Urban	19 (95.0)	535 (92.7)
Age groups, years		
16–24	8 (40.0)	152 (26.3)
25–44	5 (25.0)	257 (44.5)
45–64	5 (25.0)	143 (24.8)
65+	2 (10.0)	25 (4.3)
Gender		
Female	12 (60.0)	288 (49.9)
Male	8 (40.0)	289 (50.1)
Highest educational attainment		
University	3 (15.0)	27 (4.7)
TAFE or apprenticeship	4 (20.0)	130 (22.5)
Year 12	2 (10.0)	86 (14.9)
Year 11	5 (25.0)	103 (17.9)
Year 10	4 (20.0)	115 (19.9)
Year 9 or below	2 (10.0)	116 (20.1)
Employment status		
Full‐time	3 (15.0)	139 (24.1)
Part‐time	—	37 (6.4)
Casual	—	29 (5.0)
Work for the Dole/CDP	1 (5.0)	7 (1.2)
Other	—	4 (0.7)
None	16 (80.0)	361 (62.6)
Individual weekly income		
< $200	4 (20.0)	108 (18.7)
$200–399	5 (25.0)	150 (26.0)
$400–599	8 (40.0)	126 (21.8)
$600–799	0 (0.0)	69 (12.0)
$800+	3 (15.0)	124 (21.5)
Language spoken at home		
An Aboriginal or Torres Strait Islander language	2 (10.0)	39 (6.8)
Another language	—	8 (1.4)
English	18 (90.0)	530 (91.9)

Abbreviation: CDP, Community Development Program.

Demographics and drinking status were described for individuals who were reclassified. Median values were calculated to describe self‐reported alcohol consumption. A chi‐squared test of independence was conducted to determine whether there was a statistically significant association between drinking classification (self‐reported vs. reclassified) and the number of reported drinking occasions (1–4). Linear models were conducted to identify relationships predicting alcohol consumption amongst reclassified drinkers. Logistic regression models were conducted to identify predictors of consumption.

## 3 Results

A total of 775 Aboriginal and Torres Strait Islander peoples participated. Initially, 198 individuals said ‘no’ they did not have any alcohol in the last 12‐months. After being prompted by the check question, 20 individuals indicated that they consumed alcohol on a special occasion (Table 1). They represent 3.4% of individuals who consumed alcohol in the previous 12‐months (*n* = 20/597). They were similarly represented in the different groups of age, gender, highest educational attainment and individual weekly income, while most were not employed (Table 1).

Most reclassified drinkers self‐reported their drinking frequency as ‘once in a blue moon’ (85%; Table 2). Most reclassified drinkers reported either one or the maximum of four drinking occasions in the past year. A chi‐square test showed a significant association between the number of reported drinking occasions and drinking classification (self‐reported vs. reclassified), *χ* ^2^ (3, *n* = 597) = 27.89, *p* < 0.001, where self‐reported drinkers were more likely to report drinking on more occasions (see Table 2).

**TABLE 2:** Comparison of drinking patterns between reclassified drinkers and self‐reported current drinkers.

Variable	Reclassified drinkers, *n* = 20 (3.4%)	Self‐reported current drinkers, *n* = 577 (96.4%)
Reported drinking frequency		
Once in a blue moon	17 (85.0)	267 (46.3)
Sometimes	2 (10.0)	200 (34.7)
2–3 times a week	—	77 (13.3)
Most days	1 (5.0)	33 (5.7)
Reported drinking occasions^a^		
One occasion	6 (30.0)	31 (5.4)
Two occasions	4 (20.0)	48 (8.3)
Three occasions	2 (10.0)	23 (4.0)
Four occasions	8 (40.0)	475 (82.3)

^a^ Only a maximum of four occasions were asked about in the past year.

The reclassified drinkers reported a median consumption of 5.44 standard drinks per drinking occasion with just 0.03 median standard drinks per day across the year (Table 3). Most reclassified drinkers were in the ‘risky, but not likely dependent’ category, and the median risk category was the same for reclassified drinkers and self‐reported current drinkers (Table 3).

**TABLE 3:** Self‐reported alcohol consumption of reclassified drinkers and other drinkers.

Variable	Reclassified drinkers [Median (IQR)]	Self‐reported current drinkers [Median (IQR)]
Median standard drinks per occasion	5.44 (7.46)	8.22 (10.71)
Median standard drinks per day	0.03 (0.10)	0.31 (1.21)
Median maximum reported standard drinks	6.23 (7.04)	8.88 (10.99)
Median risk category	2.00 (0.00)	2.00 (0.00)

*Note:* Three risk categories: 1 = low‐risk, 2 = risky, not likely dependent, 3 = risky, likely dependent.

Abbreviation: IQR, interquartile range.

Using simple linear regression models (Ordinary Least Squares), we examined differences between reclassified and self‐reported drinkers on demographic and alcohol consumption characteristics. Due to the small number of reclassified drinkers, estimates are highly uncertain and should be viewed as preliminary. We found that reclassified drinkers tended to drink less frequently as reflected in lower AUDIT‐1 scores (−0.54 [95% confidence interval −0.93, −0.14], *p* = 0.008). They also reported longer stretches of consecutive days where they did not drink any alcohol (93.27 [95% confidence interval 31.6, 154.94], *p* = 0.003) in the past year relative to self‐reported drinkers (Table 4).

**TABLE 4:** Mean differences in participant characteristics and drinking patterns between reclassified and other drinkers (Ordinary Least Squares models).

Outcome	Other drinker mean [95% CI] (Intercept)	Difference (reclassified—other drinkers) [95% CI] (b)	*p*
Age	36.19 [34.98, 37.39]	−1.39 [−7.98, 5.2]	0.679
School years completed	10 [9.81, 10.19]	0.3 [−0.73, 1.33]	0.568
Frequency of drinking alcohol (AUDIT‐1)	1.79 [1.71, 1.86]	−0.54 [−0.93, −0.14]	0.008
Longest break from alcohol (days)	116.8 [105.8, 127.8]	93.27 [31.6, 154.94]	0.003
Standard drinks per occasion	13.3 [11.73, 14.87]	−4.94 [−13.5, 3.63]	0.258
Standard drinks per day	2.17 [1.54, 2.8]	−1.65 [−5.1, 1.8]	0.347
Maximum reported standard drinks	13.34 [12, 14.68]	−2.83 [−10.17, 4.5]	0.448
Risk category	2.16 [2.12, 2.2]	−0.26 [−0.47, −0.05]	0.017

*Note:* Each row represents a separate linear model. ‘Other drinker’ corresponds to the model intercept (the reference group). ‘Difference’ is the estimated difference between reclassified and self‐reported current drinkers. The intercept reflects the average outcomes for self‐reported current drinkers. The co‐efficient for reclassified drinkers indicates the difference in the outcome compared to self‐reported current drinkers. AUDIT‐1 refers to the first item of the AUDIT‐C. Risk category is an ordinal variable with three risk categories: 1 = low‐risk, 2 = risky, not likely dependent, 3 = risky, likely dependent.

Abbreviations: AUDIT, Alcohol Use Disorders Identification Test; CI, confidence interval.

There was insufficient evidence to identify demographic differences between reclassified drinkers and self‐reported current drinkers in the logistic regression models (Table 5). While there was a trend that reclassified drinkers were more likely to go to university, there was substantial variation and statistical significance of this analysis was borderline (*p* = 0.047).

**TABLE 5:** Logistic regression models of alcohol consumption characteristics and demographics among reclassified drinkers compared with self‐reported current drinkers (*n* = 597).

Outcome	Intercept (self‐reported current drinkers), OR [95% CI]	Special occasion drinker effect, OR [95% CI]	*p*
Risk category: High	19.61 [13.69, 29.34]	0.46 [0.12, 2.98]	0.312
Gender: Female	0.03 [0.01, 0.05]	1.51 [0.61, 3.89]	0.378
Employment: Full‐time	0.04 [0.03, 0.07]	0.49 [0.11, 1.49]	0.259
Highest educational attainment: TAFE/apprenticeship	0.02 [0, 0.05]	1.78 [0.34, 13.04]	0.508
Highest educational attainment: University	0.02 [0, 0.05]	6.44 [1.02, 50.76]	0.047
Individual weekly income: 200–399	0.04 [0.01, 0.09]	0.9 [0.23, 3.71]	0.877
Individual weekly income: 400–599	0.04 [0.01, 0.09]	1.71 [0.52, 6.57]	0.389
Individual weekly income: 600+	0.04 [0.01, 0.09]	0.42 [0.08, 1.94]	0.261

*Note:* Risk category: Low‐risk (category 1) versus high‐risk (categories 2 and 3, including risky but not dependent and risky, likely dependent).

Abbreviations: CI, confidence interval; OR, odds ratio.

## 4 Discussion

In this study, we aimed to determine the proportion of Aboriginal and Torres Strait Islander peoples that responded to a check question in an alcohol use survey. We found that 20 individuals (making up 3.4% of 597 individuals who reported alcohol consumption in the past 12 months) who initially self‐reported no consumption then reported alcohol consumption based on their response to a ‘check question’. We found preliminary evidence that this group of participants drank with less frequency than other drinking participants. To ensure findings are not biassed, ensuring such participants are correctly identified as drinkers is important and check questions may play a role in this.

To limit risk from alcohol consumption, National Health and Medical Research Council (NHMRC) guidelines recommend drinking no more than 4 standard drinks on any single occasion and no more than 10 standard drinks per week. In our sample, most individuals only drank episodically, but when they did, they tended to exceed the NHMRC guideline for lowering single‐occasion risk [22].

Reclassified drinkers, while still typically being at single‐occasion risk, drank less frequently, had longer consecutive day breaks from alcohol and may be more likely to be university educated (*p* = 0.047). It is not clear how to interpret this finding: does university education change how people think about being a ‘drinker’ or do university educated people in this community drink less frequently, and check questions become more relevant as people drink less frequently? However, this latter finding is particularly borderline with broad confidence intervals. This highlights that our regression findings should be interpreted with caution due to the small number of reclassified drinkers (*n* = 20). This limits our ability to generalise about reclassified drinkers. The wide confidence intervals and borderline significance for the model predicting education demonstrate that those results are exploratory and warrant further investigation in larger samples.

While we cannot be certain of the characteristics of reclassified drinkers, we have shown that they can be distinct from other drinkers and so are a source of potential bias if not detected and managed. In our sample, there were only a small number of reclassified drinkers. In our sample, without including these individuals in analyses, the proportion of individuals who did consume alcohol in the previous 12‐months is likely to be underestimated, and the typical frequency of drinking occasions is overestimated. In larger populations, or cross‐community samples, check questions may be more important in reducing bias caused by the misclassification of individuals who may drink only on special occasions [25], or who have a different conception of what it means to be a drinker.

It is unclear why some Aboriginal and Torres Strait Islander peoples initially reported not drinking. Given that they spent much of the previous 12‐months not drinking alcohol, they may have forgotten about previous drinking occasions until prompted, made errors which the check question allowed them to fix, or were initially reluctant to disclose their drinking status due to stigma and required further encouragement via the additional question [18]. Alternatively, this response may reflect how people think differently about what it means to be ‘a drinker’. Drinking status may be affected by frame‐of‐reference effects, where some may be less likely to identify as drinking alcohol if they drink less than others in their social circle. Hence, drinking self‐concept may depend in part on peer comparisons [26, 27, 28, 29]. Accordingly, check questions might be especially useful in Aboriginal communities with higher levels of alcohol consumption, as even moderate drinking in these settings may be compatible with a non‐drinking identity. Qualitative studies that explore drinking identities among Aboriginal and Torres Strait Islander peoples could provide further insight into these patterns.

Should ‘check’ questions be used in surveys among Aboriginal and Torres Strait Islander peoples? Surveys often have decision points, where responses to a single question such as ‘Do you drink alcohol?’ may dictate whether participants receive screening, or otherwise determine what items are subsequently shown. Inaccurate responses at these critical junctures can harm data completeness and validity. In the case of screening tools for drinking, inaccurate responses to items determining drinking status can mean missed opportunities to connect individuals with appropriate support to address alcohol‐related worries. Even someone who only drinks on special occasions can benefit from follow‐up as they may be at greater risk of short‐term harm [22]. This is particularly relevant in pregnancy where national guidelines recommend that there is no safe level of drinking [22]. Drinking, even on infrequent occasions during pregnancy, puts developing babies at risk of harm with potential lifelong consequences [30]. Accordingly, survey and clinical screening tool designers need to be sure that answers given at important junctures, such as those that determine drinking status, are accurate. Check questions may serve a role in doing this.

### 4.1 Strengths and Limitations

A strength of this study was that the analysis was led by a Kamilaroi and Anaiwan Aboriginal researcher and the study was developed with five other Aboriginal co‐authors. Importantly, a strength of this study included the large sample size and that the data were collected using an interactive [18], accurate [20] and acceptable [21] digital tool co‐created with Aboriginal health professionals, researchers and community leaders. This tool has been shown to be a confidential and less intimidating way to answer questions about alcohol use [31], especially in the context of trauma, stigma and fear experienced by Aboriginal and Torres Strait Islander peoples [32, 33]. Accurate and reliable data can then be used to inform community efforts to address alcohol‐related worries, improve social and emotional wellbeing, overall health [11] and to inform treatment needs, including unmet needs [34].

This study had several limitations. This study was based on self‐reported data from two Aboriginal communities located in one Australian state. There can be significant variation within and between communities and so findings may not generalise to all communities. However, given that check questions were important for these communities, and that they are quick to administer, we think they deserve more broad use so that their general utility can be better understood. The small number of reclassified drinkers made it difficult to identify characteristics of this group compared to other drinkers with precision. This is reflected in the broad confidence intervals in our regression models. Studies with larger samples would better help identify bias that failing to identify reclassified drinkers introduces. Additionally, we could not verify whether the reclassifications were accurate; therefore we cannot conclude that they improved data quality. Future studies could aim to reverify reclassifications by clinicians or by an interviewer. While this study focused on Aboriginal and Torres Strait Islander peoples, the rationale for including a check question in alcohol survey tools may also apply in general populations; however, further research with more samples taken from diverse populations would be needed to account for cultural differences.

## 5 Conclusions

We demonstrated that a check question in a digital alcohol survey tool among Aboriginal and Torres Strait Islander peoples was useful to identify individuals who initially self‐reported no alcohol consumption, but then said they did drink on special occasions. This finding underscores the importance of incorporating questions about alcohol use during special or culturally significant occasions—such as Christmas, New Year, sporting events or periods of ‘sorry business’—to capture more accurate consumption data. When healthcare and national surveys rely on alcohol survey tools that overlook alcohol consumption that occurs on special occasions, valuable data may be missed and opportunities to connect individuals with culturally appropriate support may be lost. Accurate drinking estimates are important to inform the needs of services for Aboriginal and Torres Strait Islander peoples, including strategies to prevent and address alcohol‐related worries.

## Author Contributions

Each author certifies that their contribution to this work meets the standards of the International Committee of Medical Journal Editors.

## Funding

This work is supported by the Australian National Health and Medical Research Council through a Project Grant (#1087192) and an Ideas Grant (#1183744); and via the Medical Research Future Fund via a Primary Health Care and Digital Innovation Grant (#MRF2021660). We are grateful to the local research team and study communities who participated in this study.

## Conflicts of Interest

The authors declare no conflicts of interest.

## Data Availability

Research data are not shared due to ethical restrictions.

## References

[1] P. Dudgeon , M. Wright , Y. Paradies , D. Garvey , and I. Walker , “Aboriginal Social, Cultural and Historical Contexts,” in Working Together: Aboriginal and Torres Strait Islander Mental Health and Wellbeing Principles and Practice, 2nd ed., ed. P. Dudgeon , H. Milroy , and R. Walker (Australian Government Department of Health and Ageing, 2014), 25–42.

[2] S. A. Hunter , H. Skouteris , and H. Morris , “A Conceptual Model of Protective Factors Within Aboriginal and Torres Strait Islander Culture That Build Strength,” Journal of Cross‐Cultural Psychology 52, no. 8–9 (2021): 726–751, 10.1177/00220221211046310.

[3] L. Pulver , M. Haswell , I. Ring , et al., “Indigenous Health‐Australia, Canada, Aotearoa New Zealand, and the United States‐Laying Claim to a Future That Embraces Health for Us All,” World Health Report (2010). Background paper 33, http://www.who.int/healthsystems/topics/financing/healthreport/33IH.pdf.

[4] G. T. Nadew , “Exposure to Traumatic Events, Prevalence of Posttraumatic Stress Disorder and Alcohol Abuse in Aboriginal Communities,” Rural and Remote Health 12, no. 4 (2012): 1667. 23072253

[5] M. Gracey and M. King , “Indigenous Health Part 1: Determinants and Disease Patterns,” Lancet 374, no. 9683 (2009): 65–75, 10.1016/S0140-6736(09)60914-4. 19577695

[6] S. Harradine , “My Version—The Stolen Generation 1945–1952,” Ngoonjook: Journal of Australian Indigenous Issues 9 (1993): 1–4.

[7] HealingFoundation , “Stolen Generations,” https://healingfoundation.org.au/stolen‐generations/. (accessed 27 June 2026).

[8] L. Holland , N. Reid , N. Hewlett , et al., “Alcohol Use in Australia: Countering Harm With Healing,” Lancet Regional Health—Western Pacific 37 (2023): 100774, 10.1016/j.lanwpc.2023.100774. 37693874 PMC10485668

[9] Australian Institute of Health and Welfare, National Indigenous Australians Agency , Measure 2.16 Risky Alcohol Consumption (Australian Institute of Health and Welfare and National Indigenous Australians Agency, 2023), https://www.indigenoushpf.gov.au/measures/2‐16‐risky‐alcohol‐consumption.

[10] R. Gray , “Shame, Labeling and Stigma: Challenges to Counseling Clients in Alcohol and Other Drug Settings,” Contemporary Drug Problems 37, no. 4 (2010): 685–703.

[11] K. S. K. Lee , T. Chikritzhs , S. Wilson , et al., “Better Methods to Collect Self‐Reported Alcohol and Other Drug Use Data From Aboriginal and Torres Strait Islander Australians,” Drug and Alcohol Review 33, no. 5 (2014): 466–472, 10.1111/dar.12159. 24849378

[12] P. d'Abbs and N. Hewlett , “Explaining Aboriginal Alcohol Use: Changing Perspectives, Hidden Assumptions,” in Learning From 50 Years of Aboriginal Alcohol Programs: Beating the Grog in Australia, ed. P. d'Abbs and N. Hewlett (Springer Nature Singapore, 2023), 17–54.

[13] J. H. Conigrave , S. Wilson , K. M. Conigrave , et al., “Countering Stereotypes: Exploring the Characteristics of Aboriginal Australians Who Do Not Drink Alcohol in a Community Representative Sample,” Drug and Alcohol Review 43 (2024): 1523–1533, 10.1111/dar.13907. 39042571

[14] K. S. K. Lee , J. H. Conigrave , S. Wilson , et al., “Deeper Understandings of Patterns of Drinking Among Aboriginal and Torres Strait Islander Australians: Informing Policy and Practice,” Health Promotion Journal of Australia 34, no. 4 (2023): 883–888, 10.1002/hpja.696. 36740591 PMC10946760

[15] E. M. Hunter , W. D. Hall , and R. M. Spargo , “Patterns of Alcohol Consumption in the Kimberley Aboriginal Population,” Medical Journal of Australia 156, no. 11 (1992): 764–768. 1352843 10.5694/j.1326-5377.1992.tb121557.x

[16] J. Fleming , C. Watson , D. McDonald , and K. Alexander , “Drug Use Patterns in Northern Territory Aboriginal Communities 1986–1987,” Drug and Alcohol Review 10, no. 4 (1991): 367–380. 16818300 10.1080/09595239100185421

[17] K. S. K. Lee , M. S. Fitts , J. H. Conigrave , et al., “Recruiting a Representative Sample of Urban South Australian Aboriginal Adults for a Survey on Alcohol Consumption,” BMC Medical Research Methodology 20, no. 1 (2020): 183, 10.1186/s12874-020-01067-y. 32631364 PMC7339418

[18] K. S. K. Lee , S. Wilson , J. Perry , et al., “Developing a Tablet Computer‐Based Application (‘App’) to Measure Self‐Reported Alcohol Consumption in Indigenous Australians,” BMC Medical Informatics and Decision Making 18, no. 1 (2018): 8, 10.1186/s12911-018-0583-0. 29334962 PMC5769490

[19] K. S. K. Lee , J. H. Conigrave , S. Callinan , et al., “Asking About the Last Four Drinking Occasions on a Tablet Computer as a Way to Record Alcohol Consumption in Aboriginal and Torres Strait Islander Australians: A Validation,” Addiction Science & Clinical Practice 14, no. 1 (2019): 15. 31039824 10.1186/s13722-019-0148-2 PMC6492339

[20] K. S. K. Lee , J. H. Conigrave , S. Wilson , et al., “Short Screening Tools for Risky Drinking in Aboriginal and Torres Strait Islander Australians: Modified AUDIT‐C and a New Approach,” Addiction Science & Clinical Practice 14, no. 1 (2019): 22. 31256762 10.1186/s13722-019-0152-6 PMC6600888

[21] K. S. K. Lee , J. H. Conigrave , M. Al Ansari , et al., “Acceptability and Feasibility of a Computer‐Based Application to Help Aboriginal and Torres Strait Islander Australians Describe Their Alcohol Consumption,” Journal of Ethnicity in Substance Abuse 20 (2021): 16–33. 30887909 10.1080/15332640.2019.1579144

[22] National Health and Medical Research Council , Australian Guidelines to Reduce Health Risks From Drinking Alcohol. Commonwealth of Australia (National Health and Medical Research Council, 2020).

[23] T. Alanko , “An Overview of Techniques and Problems in the Measurement of Alcohol Consumption,” in Research Advances in Alcohol and Drug Problems, ed. G. S. Reginald , D. C. Howard , B. G. Frederick , et al. (Springer, 1984), 209–226.

[24] R Core Team , R: A Language and Environment for Statistical Computing (Foundation for Statistical Computing, 2021).

[25] T. Stockwell , S. Donath , M. Cooper‐Stanbury , T. Chikritzhs , P. Catalano , and C. Mateo , “Under‐Reporting of Alcohol Consumption in Household Surveys: A Comparison of Quantity‐Frequency, Graduated‐Frequency and Recent Recall,” Addiction (Abingdon, England) 99, no. 8 (2004): 1024–1033, 10.1111/j.1360-0443.2004.00815.x. 15265099

[26] H. W. Marsh , “Self‐Concept: The Application of a Frame of Reference Model to Explain Paradoxical Results,” Australian Journal of Education 28, no. 2 (1984): 165–181, 10.1177/000494418402800207.

[27] G. H. Bodkin‐Andrews , A. Dillon , and R. G. Craven , “Bangawarra'gumada—Strengthening the Spirit: Causal Modelling of Academic Self‐Concept and Patterns of Disengagement for Indigenous and Non‐Indigenous Australian Students,” Australian Journal of Indigenous Education 39, no. 1 (2010): 24–39, 10.1375/S1326011100000892.

[28] Oxford Academic , “Social Norm Influences on Evaluations of the Risks Associated with Alcohol Consumption: Applying the Rank‐Based Decision by Sampling Model to Health Judgments | Alcohol and Alcoholism,” https://academic.oup.com/alcalc/article‐abstract/47/1/57/216119?redirectedFrom=fulltext. 10.1093/alcalc/agr146 22101815

[29] D. M. Litt , M. A. Lewis , H. Stahlbrandt , P. Firth , and C. Neighbors , “Social Comparison as a Moderator of the Association Between Perceived Norms and Alcohol Use and Negative Consequences Among College Students,” Journal of Studies on Alcohol and Drugs 73, no. 6 (2012): 961–967. 23036214 10.15288/jsad.2012.73.961 PMC3469049

[30] Australian FASD Guidelines Development Group , Australian Guidelines for Assessment and Diagnosis of Fetal Alcohol Spectrum Disorder (FASD Hub Australia, 2025), https://fasdhub.org.au/fasd‐information/australian‐guidelines‐for‐assessment‐and‐diagnosis‐of‐fasd/.

[31] K. K. Lee , J. H. Conigrave , E. Dale , et al., “Acceptability and Quality of the “Grog Survey App” Brief Intervention: Helping Aboriginal Australians Reflect on Their Drinking Using a Digital Health Tool,” Drug and Alcohol Review 44 (2024): 119–132, 10.1111/dar.13964. 39449109

[32] M. M. Islam , H. T. Oni , K. S. K. Lee , et al., “Standardised Alcohol Screening in Primary Health Care Services Targeting Aboriginal and Torres Strait Islander Peoples in Australia,” Addiction Science & Clinical Practice 13, no. 1 (2018): 5, 10.1186/s13722-018-0108-2. 29592801 PMC5875000

[33] K. S. K. Lee , B. Freeburn , S. Ella , W. Miller , J. Perry , and K. M. Conigrave , Handbook for Aboriginal Alcohol and Drug Work (University of Sydney, 2012), 464.

[34] J. Brett , K. S. K. Lee , D. Gray , et al., “Mind the Gap: What Is the Difference Between Alcohol Treatment Need and Access for Aboriginal and Torres Strait Islander Australians?,” Drug and Alcohol Review 35, no. 4 (2016): 456–460, 10.1111/dar.12313. 26331675

